# SpecReFlow: an algorithm for specular reflection restoration using flow-guided video completion

**DOI:** 10.1117/1.JMI.11.2.024012

**Published:** 2024-04-24

**Authors:** Haoli Yin, Rachel Eimen, Daniel Moyer, Audrey K. Bowden

**Affiliations:** a Vanderbilt University, Department of Computer Science, Nashville, Tennessee, United States; b Vanderbilt University, Vanderbilt Biophotonics Center, Nashville, Tennessee, United States; c Vanderbilt University, Department of Biomedical Engineering, Nashville, Tennessee, United States; d Vanderbilt University, Department of Electrical and Computer Engineering, Nashville, Tennessee, United States

**Keywords:** specular reflection, image artifacts, image restoration, optical flow, multiview restoration, endoscopy

## Abstract

**Purpose:**

Specular reflections (SRs) are highlight artifacts commonly found in endoscopy videos that can severely disrupt a surgeon’s observation and judgment. Despite numerous attempts to restore SR, existing methods are inefficient and time consuming and can lead to false clinical interpretations. Therefore, we propose the first complete deep-learning solution, SpecReFlow, to detect and restore SR regions from endoscopy video with spatial and temporal coherence.

**Approach:**

SpecReFlow consists of three stages: (1) an image preprocessing stage to enhance contrast, (2) a detection stage to indicate where the SR region is present, and (3) a restoration stage in which we replace SR pixels with an accurate underlying tissue structure. Our restoration approach uses optical flow to seamlessly propagate color and structure from other frames of the endoscopy video.

**Results:**

Comprehensive quantitative and qualitative tests for each stage reveal that our SpecReFlow solution performs better than previous detection and restoration methods. Our detection stage achieves a Dice score of 82.8% and a sensitivity of 94.6%, and our restoration stage successfully incorporates temporal information with spatial information for more accurate restorations than existing techniques.

**Conclusions:**

SpecReFlow is a first-of-its-kind solution that combines temporal and spatial information for effective detection and restoration of SR regions, surpassing previous methods relying on single-frame spatial information. Future work will look to optimizing SpecReFlow for real-time applications. SpecReFlow is a software-only solution for restoring image content lost due to SR, making it readily deployable in existing clinical settings to improve endoscopy video quality for accurate diagnosis and treatment.

## 1 Introduction

Specular reflection (SR) is an optical phenomenon wherein light is strongly reflected from a surface in a unified direction based on Snell’s law. On a camera, SR often leads to saturation of the pixels (causing them to appear white) and is well known for obscuring the underlying information in the image—rendering it a functional blind spot. In clinical settings, that blind spot can critically impair all three stages of surgical operations: diagnosis, planning, and postoperative observation. Moreover, SR artifacts impact the interpretation of clinical endoscopy data and can hinder the utility of augmented/virtual reality (AR/VR) tools for surgical navigation and the creation of accurate three-dimensional (3D) organ models that have been found to improve surgical outcomes.^1^^–^^3^ Thus, there is a significant positive impact associated with improving video quality by restoring SR regions. Several studies have directly linked enhanced image restoration to improved performance in downstream medical tasks. For instance, Ali et al.^4^ found that the restoration of SR and saturated regions significantly improved image feature matching, which could be used for 3D organ reconstruction for further analysis. This and other studies^5^^,^^6^ collectively suggest that advancements in image restoration quality not only are beneficial in their own right but also play a crucial role in enhancing the accuracy and effectiveness of various medical applications.

We propose a novel method for accurate, multiview restoration of SR regions of endoscopy data based on the propagation of optical flow^7^ from adjacent frames of a video sequence. Optical flow is defined as the pattern of apparent movement of objects in a visual scene caused by relative motion between an observer and a scene. The optical flow concept has been commonly applied to tasks such as object detection and tracking, movement detection, robot navigation, and visual odometry;^8^ however, we are the first to apply it to SR restoration of medical endoscopy data. The optical flow method that we employ utilizes pixel movement data, enabling a transformer deep learning model to effectively integrate pixel information into the SR region. Pretrained on various video data, this model is proficient at adapting to different lighting conditions in SR regions. Its adaptability is crucial for maintaining spatiotemporal consistency in SR regions informed by optical flow. The flow-guided transformer (FGT) algorithm^9^ that we use implicitly accounts for depth information via optical flow, which aids in lighting adjustments during the restoration process. As a result, the restored pixels in the SR regions are not merely copied from another frame; their colors are also appropriately transformed. Intuitively, this method preserves the texture of the restored image content while allowing color to adapt seamlessly to its new background. We evaluate the effectiveness of our method in the context of other state-of-the-art restoration strategies and propose new assessment metrics appropriate for this task, termed mPSNR and mSSIM, to evaluate free-form SR and masked regions.

The ability to remove and restore SR regions in an accurate and color-consistent manner is useful for improving the potential for both accurate analysis and diagnosis of endoscopy images. Our algorithm represents a major contribution to the field by being the first full system to combine both temporal and spatial information from endoscopy videos for effective detection and restoration of SR regions, surpassing previous methods that rely solely on spatial information. Importantly, unlike other work for optical flow applied to SR restoration,^10^ our implementation is a software-only solution that does not require additional equipment beyond the clinical standard of care for endoscopy, making it readily deployable for clinical use.

## 2 Related Works

One strategy to eliminate SR artifacts is to replace them with new information that infers the original content. Efforts to restore the appearance of the clinical data underlying SR artifacts can be broadly categorized into three groups: single-frame conventional methods, single-frame learning methods, and multi-frame matching methods.

Single-frame conventional methods include traditional image processing methods such as thresholding or filtering. One limitation of using traditional techniques for detecting SR is that it may result in some SR regions—especially smaller regions—going undetected, which prevents restoration.^11^^–^^13^ In addition, conventional methods for SR restorations largely depend on empirical parameter setting, which can lead to slow runtimes and inaccurate feature appearance such as blurriness, structural inaccuracy, and poor color blending with the surrounding non-SR region.^6^^,^^11^^–^^17^

Many single-frame methods use deep learning approaches, such as convolutional neural networks (CNNs)^18^^,^^19^ or generative adversarial networks,^4^^,^^5^^,^^20^^–^^22^ to directly inpaint the region for restoration. Although learned methods may lead to fast and high-quality restorations, the generated regions may not be truly representative of the ground-truth tissue, as they only inpaint the missing SR region using local spatial information obtained from a single perspective.

Multi-frame matching methods involve the use of multiple frames (e.g., video datasets). SR regions can be restored by directly replacing SR pixels that are present in one frame with the corresponding pixels of the same region that are not covered by SR in another frame. This is possible because SR can be thought of as a dynamic object with no apparent pattern of movement. SR regions vary among frames due to the varying angle of incidence of the light source, which may be caused by changes in the camera angle relative to the tissue, changes in the smoothness of the tissue, or other variations in the optical density of the intervening medium. These factors affect how light is reflected off the surface, leading to changes in the appearance and location of SRs in each frame. A significant drawback of simple replacement approaches (e.g., homography transform), however, is that the restored SR regions do not have the same brightness of color as the surrounding area due to lighting inconsistencies caused by camera or light source movement from one frame to another. The result of these lighting aberrations is seen as temporal inconsistency and color flickering.^23^^,^^24^ Recent efforts, such as those by Daher et al.,^25^ have tried to harness temporal data using traditional nonlearning methods to detect SR regions. However, their limited accuracy in SR detection causes temporal-aware generative adversarial models to unintentionally include undetected SR from other regions, resulting in noisy reconstructions.

Finally, a general limitation of all previous specular restoration studies performed to date is that the standard metrics used to evaluate the effectiveness of the restoration techniques [e.g., peak signal-to-noise ratio (PSNR) and structural similarity metric (SSIM)] have settings (e.g., kernel size) that are often not fully explained or standardized, which can make it difficult to accurately compare the results of different methods. Furthermore, as we will show, these standard metrics are not sensitive enough to capture important details relevant to the scope of the restoration task, or they may not be applied to the most relevant regions of the images being restored. As a result, it is difficult to accurately assess the true improvement in image quality achieved by existing methods.

## 3 Methodology

### 3.1 Overview

**Fig. 1:**
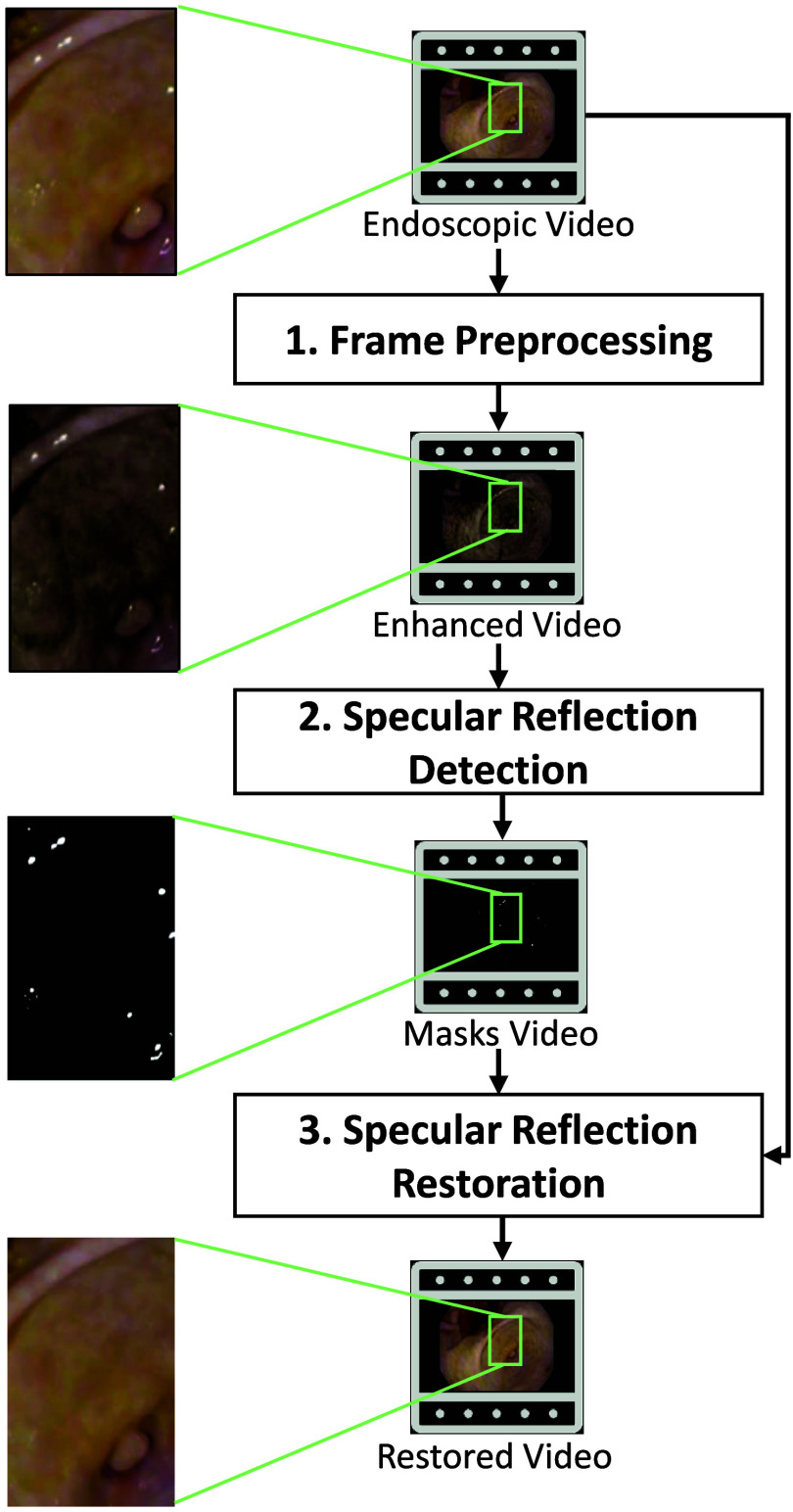
Simplified overview of the SpecReFlow algorithm. A white-light endoscopy video is used as input; it is then preprocessed to create an enhanced video for input to the detection stage for better results. After the detection stage, SR binary masks are generated for each video frame, and both the original endoscopic video and the mask video are used during the restoration stage to create the restored video.

SpecReFlow is an SR detection and restoration algorithm with three stages: (1) image preprocessing, (2) SR detection, and (3) SR restoration. Figure 1 presents an overview of the framework. The preprocessing stage increases the contrast of SR in the original image to simplify the detection task. The SR detection stage combines a modified U-net model that detects small SR regions with a thresholding method to detect the larger SR regions on which deep learning methods commonly fail. The SR restoration stage leverages recent progress in flow-based video completion using optical flow and vision transformers to distinguish the dynamic foreground image (SR) from the dynamic background image (ground truth tissue)^26^ and inpaint the detected SR regions. All work was implemented in Python 3.10 using PyTorch version 1.13.1+cu117 with Ubuntu 22.04 on an NVIDIA GeForce RTX 3090 GPU with 24 GB VRAM, 32 GB RAM, and an AMD Ryzen 9 5950X with 16 logical processors.

### 3.2 Dataset

For model training and pipeline development, we used the CVC-EndoSceneStill from Vázquez et al.,^27^ a compilation of 912 frames obtained from 44 endoscopy videos from 36 patients. We chose this dataset because it is one of the only known datasets with manually annotated SR region masks.

To increase the amount of training data for the deep learning detection model, we employed data augmentation techniques: each image was cropped into four equal-sized, smaller images of 288×384  pixels and subjected to horizontal/vertical flips at random. The order of the entire dataset was then randomized to ensure sample independence. The train/validation/test split was 80/10/10 based on common splits in other papers.^4^^,^^19^

### 3.3 Algorithm Design

#### 3.3.1 Stage 1: image preprocessing

**Fig. 2:**
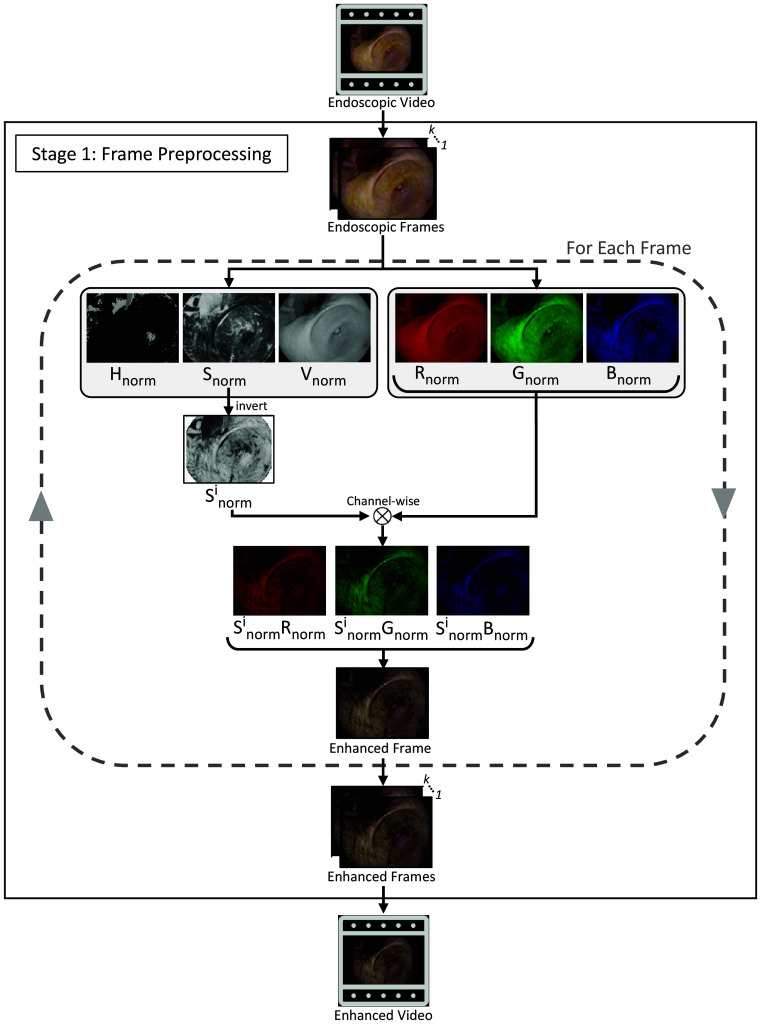
Image preprocessing stage visualization. A white-light endoscopy video is used as input, and each video frame is processed to create an enhanced video. For each frame, HSV and RGB images are created. Then, the saturation (S) channel is inverted through a 1-S operation and multiplied with each channel of the RGB image to ultimately create the enhanced frame.

To improve the contrast between SR regions and surrounding regions, we used an image enhancement algorithm proposed by Saint-Pierre et al.^11^ that modifies the pixel intensity histogram distribution by multiplying the red, green, and blue (RGB) planes with the 1-S plane of the hue, saturation, and value (HSV) color model, as shown in Fig. 2. When observing the distribution of pixel intensities, we found that this step leads to an increased gap between the SR values and the rest of the histogram, which makes it easier to separate SR and non-SR regions in the detection stage.

#### 3.3.2 Stage 2: SR detection

**Fig. 3:**
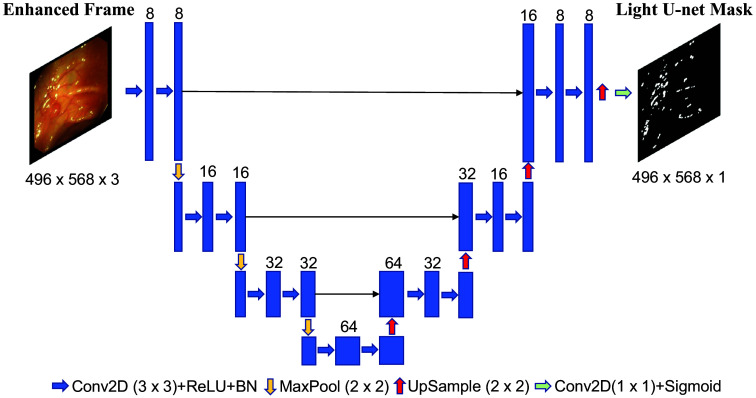
Architecture of our Light U-net model. We use four layers and eight initial convolutional filters to create an efficient and optimized architecture as determined by a neural architecture search.

Our SR detection algorithm combines deep learning with a traditional thresholding algorithm. The deep learning detection model, which we call “Light U-net,” is built upon the popular and proven U-net architecture for biomedical segmentation tasks.^28^ The original U-net model^28^ included 64 convolutional layers and went five layers deep (i.e., it had four downsampling operations on the encoder side). We use only four layers (with three downsampling operations on the encoder side) and eight convolutional layers, yielding a total of 121,641 trainable parameters. The resulting model, shown in Fig. 3, is lighter and more efficient for SR detection. We used fewer layers because a smaller model allowed for less complexity to detect relatively simple objects such as SR. In addition, the reduced complexity of the model allows for real-time SR detection, which can be combined with an appropriate SR restoration algorithm to fix SRs in real time.

To train our Light U-net model, we used a combination of Dice loss and binary cross entropy loss, a U-net four levels deep (i.e., three pooling operations), an Adam optimizer (initial learning rate of 0.01, $\beta_{1}=0.9$, $\beta_{1}=0.999$), and a learning rate scheduler of ReduceLrOnPlateau (patience = 10, factor = 0.1). These settings were then used to train the model on 120 epochs with a batch size of eight. We found that training the model on more epochs led to overfitting and a decreased performance on the validation and test sets.

On its own, the Light U-net model learns to detect smaller SR regions significantly better than larger regions, which can be explained in two ways. First, the training dataset was relatively unbalanced as there were significantly more samples of smaller, scattered SRs; thus, the model often misidentified larger SR regions as non-SR objects. Second, the fixed receptive field of the convolutional kernels caused the model to learn more of the smaller SR regions. Because the training frames were of a lower resolution than true endoscopy frames, the model was primed to detect smaller SR regions in higher-resolution frames.

Hence, to enable the detection of large SR regions, we utilized a simple thresholding method. It is well known that SR regions have higher pixel intensity values than surrounding regions based on the thresholding algorithm proposed by Yu et al.^29^ and the analysis of a histogram of pixel intensities within a frame with SR. We chose the threshold I(x,y)>194, where I(x,y) is the pixel intensity in the value channel of the HSV image and (x,y) is the location of the pixel, by optimizing the detection metrics [i.e., Dice score and intersection over union (IoU)] through a greedy search on the same training set used for U-net training. Although this basic method worked well on large SR regions, our testing (as shown in Sec. 4.1) revealed that thresholding alone is insufficient for detecting all SR as it misses relatively low-intensity SR commonly found in smaller SR regions. Inspired by Xu et al.,^30^ to harness the strength of both algorithms, our final strategy was a combination of deep learning mask prediction (good at small, scattered SR) and thresholding mask prediction (good at large SR) through an AND operation of the masks generated by each prediction method. In summary, the final, optimal SR detection stage algorithm that we adopted is the Light U-net + thresholding algorithm, with an additional dilation step that we describe and justify in Sec. 4.1.

#### 3.3.3 Stage 3: SR restoration

In this work, we chose to use an existing flow-based algorithm for the SR restoration stage. We tested three state-of-the-art algorithms for flow-based video completion to determine which performs best for our task: (1) the Flow edge-Guided Video Completion (FGVC) algorithm from 2020,^26^ modified by us to use the state-of-the-art single-frame inpainting model, LaMa,^31^ instead of DeepFillv1 to test more recent methodologies; (2) the End-to-End Flow-Guided Video Inpainting (E2FGVI) algorithm,^32^ which comprises three main modules—flow completion, feature propagation, and content hallucination—all tied together in a streamlined manner to allow for fast, efficient, and accurate restoration of SR regions; (3) the FGT,^9^ which improves upon FGVC by replacing the edge completion part of flow estimation with an edge loss function in a novel flow estimate network, mitigating additional computational complexity. After color propagation along the estimated flow, FGT completes the missing pixels of multiple frames at once using a spatial and temporal transformer model, incorporating more information to complete missing pixels more efficiently than the single-frame and iterative inpainting version of FGVC. To compare these flow-based models against a baseline, we also analyzed two single-frame inpainting methods representing the current standard in SR restoration: LaMa^31^ and DeepFillv2.^33^

**Fig. 4:**
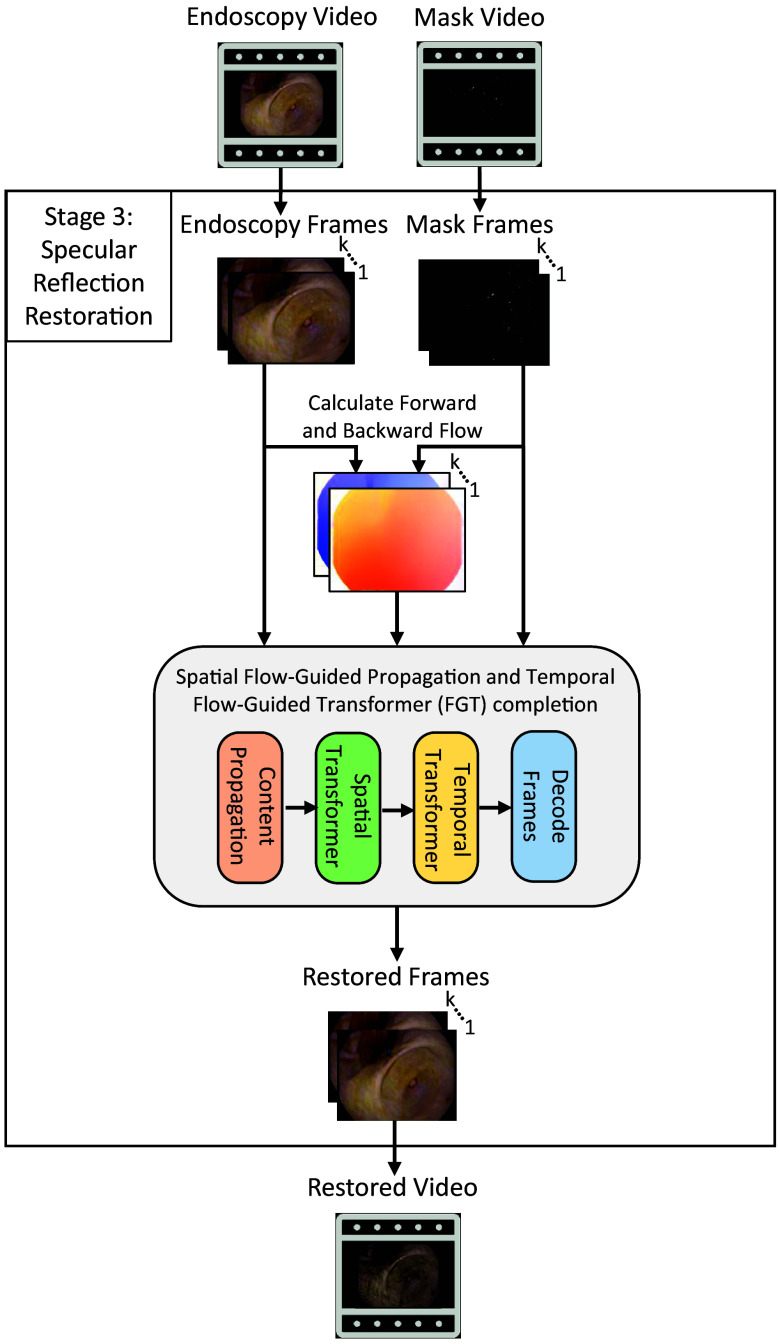
SR restoration stage visualization. The original endoscopy video and SR binary masks generated from the SR detection stage are used as inputs. Once frames are sampled from the video, forward and backward flows are calculated for adjacent frames, and all three datasets are used as inputs to the flow propagation and the FGT, which performs frame restoration. The restored frames are then combined to synthesize the restored video.

Based on the experimentation and discussion in the following sections, we determined that FGT accomplished the optical flow-based restoration task better than the other two. Hence, SpecReFlow uses FGT as its third stage; an overview of the restoration algorithm is given in Fig. 4.

### 3.4 Evaluation Strategy and Metrics

#### 3.4.1 SR detection

To test the effectiveness of the SR detection stage, we reserved ∼10% of the CVC-EndoSceneStill dataset detailed in Sec. 3.2 (separate from the other 90% utilized for training), which was 184 images. The following quantitative metrics were evaluated: sensitivity, Jaccard/IoU score, and Dice score. Here, we define sensitivity to be the number of true-positive SR pixels detected divided by the total number of SR pixels in an image. Sensitivity, rather than specificity, is relevant because our end goal is not detection but restoration; hence, we recognize that the detected SR mask does not have to be exact to the pixel (i.e., high specificity), but rather needs to cover as many true-positive regions as possible. Note that using sensitivity alone will give a false impression of the performance as over-detection (of non-SR pixels) can also lead to high sensitivity; thus, we first optimized the detection algorithm for high IoU and Dice scores to limit the detected regions to locations of true SR pixels. Our qualitative analysis sought to validate whether our method masks all SR regions and any surrounding regions affected by increased saturation. In both cases, we compared our results to baselines of existing, open-source SR region-detection methods: a conventional histogram thresholding method by Tchoulack et al.,^34^ a more recent non-deep learning adaptive robust principal component analysis (RPCA) method,^16^ and a denoising convolutional neural network (DnCNN) modified to detect SR regions by Zhang et al.^35^ We chose these methods because they represent the broad range of approaches taken to solve the detection problem in the past and are the current state-of-the-art methods for open-source implementations.

#### 3.4.2 SR restoration

To identify which SR restoration method performs best for endoscopy data, we generated a synthetic dataset. We overlaid the GLENDA dataset (360×640-pixel resolution) from Leibetseder et al.,^36^ which contains over 13,000 unannotated (no SR labels/masks), nonpathological images from 20+ laparoscopy endometriosis video surgeries, with synthetic SR masks pulled from the CVC-EndoSceneStill dataset and resized to the appropriate dimensions. The latter dataset contained 200 masks, from which 50 independent random masks were chosen to be applied to frames as described in the following experimental procedure. Note that SR masks were not applied to regions of GLENDA frames that had pre-existing SR.

We chose to test three flow-based algorithms, FGVC, FGT, and E2FGVI, along with two single-frame inpainting methods, DeepFillv2 and LaMa, as these were the state-of-the-art algorithms for their respective categories. Each experimental trial comprised an independent set of 100 sequential frames, defined as a sequence of video frames that do not overlap temporally with another set of 100 sequential frames. To provide the flow-based algorithms with sufficient temporal data (not relevant for the single-frame inpainting algorithms as they only require spatial data), the first 50 frames were considered “warm-up” data, and the following 50 frames were used for “assessment” for both classes of algorithms. No masks were applied to the “warm-up” dataset. To account for the potential spatial overlap (i.e., footage circles back to the same region) between temporally independent sets of frames, the process of selecting random masks also allowed for different regions to be restored despite the overlap, preserving the spatial independence of each frame sequence. In total, 13 independent restoration trials were conducted for each algorithm.

**Fig. 5:**
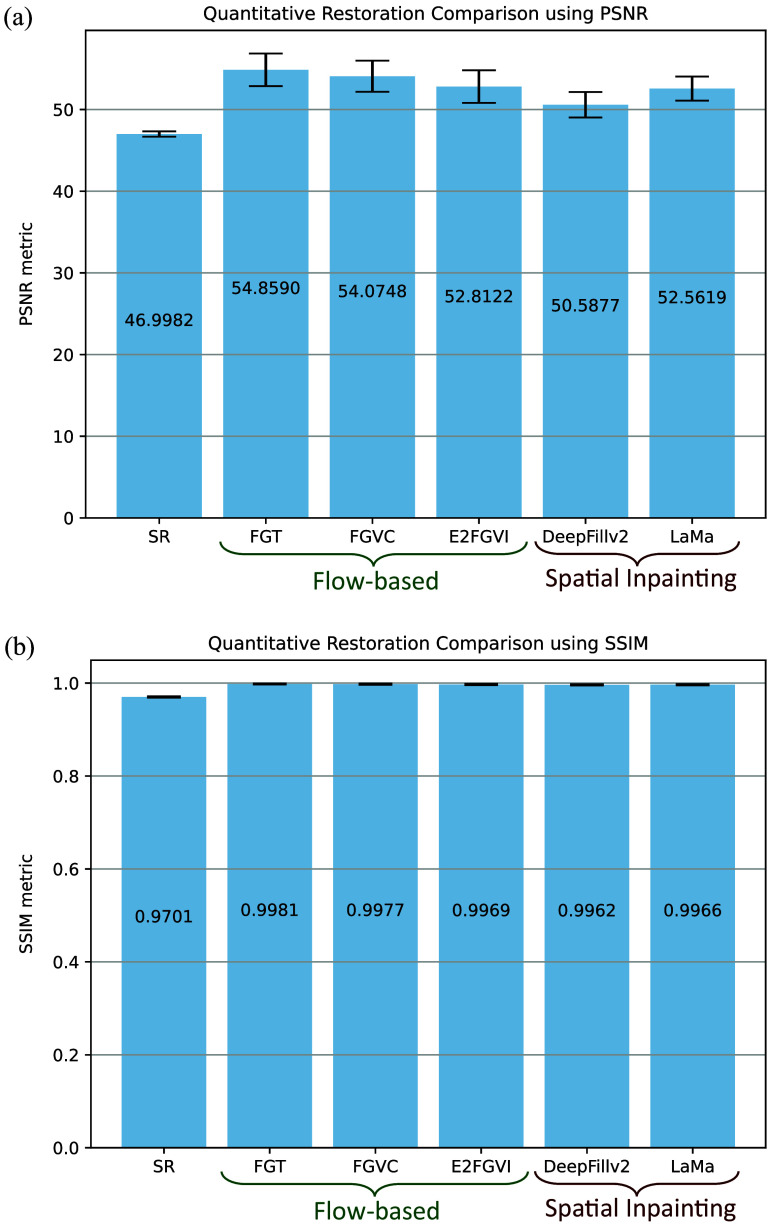
Original restoration metrics for quantitative restoration analysis. (a) PSNR and (b) SSIM performance of various restoration algorithms show insignificant differences. The following restoration algorithms and baselines are compared: SR unrestored image as the baseline; optical flow-based methods such as FGT, FGVC, and E2FGVI; and single-frame inpainting methods such as DeepFillv2 and LaMa.

Our metrics of assessment were modified versions of the established PSNR and the SSIM, which we term mPSNR and mSSIM, respectively. The need for modifications stems from the specifics of our use case. In general, PSNR is used as a quality measurement to indicate how well the restored image compares to the fidelity of the original image. When applied to evaluate the effectiveness of various restoration algorithms, we observed that the difference in PSNR outcomes [Fig. 5(a)] is statistically insignificant (p>0.05 in a two-sample t-test); we believe that this result is due to the introduction of excess noise from non-SR regions included in the PSNR calculation. Similarly, SSIM measures perceived changes in structural information. Because SSIM uses a sliding window/kernel across the entire image, the resulting structural similarities are nearly indistinguishable for the various algorithms and are close to ideal across all restoration methods, as shown in Fig. 5(b). We believe that this result is due to the relatively small size of SR regions compared with the image size. Hence, we observed that the original formulas for PSNR and SSIM do not fit our use case well to distinguish restoration algorithms.

We thus modified the metrics as follows. Our proposed mPSNR differs from PSNR in that we zero out the error terms for non-SR pixels and replace the standard mean square error (MSE) term with a modified MSE in which we divide the MSE by the number of SR pixels instead of the total number of pixels in the frame. The overall result is that only SR pixels from the image contribute to the metric assessment. The formula to describe the mPSNR between the ground truth and a restored image is

$$\mathrm{mPSNR}=20*\log(\frac{{\mathrm{MAX}}_{I}}{\sqrt{\mathrm{mMSE}}}),$$ (1)

$$\mathrm{mMSE}=\frac{1}{C}\frac{1}{S}\overset{m-1}{\underset{i=0}{\sum }}\overset{n-1}{\underset{j=0}{\sum }}{\left[I(i,j)-K(i,j)\right]}^{2}*w,$$ (2)

where C is the number of color channels, S is the number of SR region pixels, I is the original image of size m×n, K is the restored image, i and j are pixel indexes, and w is a binary weight, which is 1 for SR pixels and 0 for non-SR pixels. For Eq. (1), MAX is the maximum possible pixel value of the image, which is 255 for an 8-bit image and 1.0 for a normalized image.

Our mSSIM also provides focus on the SR regions rather than the entire frame as this change better captures structural inconsistencies of the restored regions. Because SSIM requires rectangular regions, we first extracted bounding boxes from the ground truth SR masks. We chose a minimum bounding box size of eight to facilitate the use of sliding windows (of size seven) during the SSIM operation. Once the SSIM of each bounding box was found, the frame-level mSSIM was computed as a weighted average of the number of SR pixels in a particular bounding box divided by the total number of SR pixels in the frame. Note that the possible ranges of values for these metrics are still the same as that of the unmodified versions, i.e. [0, unbounded) for mPSNR and [0, 1] for mSSIM. The algorithm is detailed in Algorithm 1.

**Algorithm 1:** mSSIM calculation

**Require:** Ground Truth Image (origImg), Restored Image (restoredImg) and SR Mask
**Ensure:** mSSIM Metric
1: Initialize ssims, weights = [ ], [ ]
2: boundingBoxes = Get bounding boxes for each separate SR region in SR Mask
3: **for** bBox in boundingBoxes **do**
4: Initialize boxHeight, boxWidth
5: **if** boxHeight <8 **then**
6: add to each side until minimum height of sliding window dimension
7: **end if**
8: **if** boxWidth <8 **then**
9: add to each side until minimum width of sliding window dimension
10: **end if**
11: ssim = Calculate ssim with origImg[bBox], restoredImg[bBox], windowSize=7
12: ssims.append(ssim)
13: weights.append(sum(mask[bBox]))
14: **end for**
15: totalSpecularReflections = sum(weights)
16: weights = weights/totalSpecularReflections
17: **return** weights×ssims

## 4 Results

### 4.1 SR Detection

The results reported in Table 1 reveal that our initial detection algorithm (SpecReFlow Det(-)) achieved Dice score and IoU values that were more than 1.17× and 1.22× higher, respectively, than the next best established method (modified DnCNN), and it was nearly 4.25× faster. As observed, SpecReFlow detection performs favorably against existing SR detection algorithms of all types in both measures of effectiveness and efficiency.

**Table 1:** Quantitative comparison of SR detection metrics.

Detection method	Dice ↑	IoU ↑	Sensitivity ↑	Time (ms) ↓
Tchoulack algorithm^34^	0.5012	0.3500	0.5122	1212
Adaptive RPCA^16^	0.6094	0.4590	0.5960	**3.70**
Modified DnCNN^35^	0.7096	0.5853	0.6210	53.17
SpecReFlow Det. (-)	**0.8285**	**0.7158**	0.8158	11.06
SpecFeFlow Det.	0.6517	0.4947	**0.9457**	11.32

Note: bold values highlight the best performing algorithm for each metric.

Although our initial SpecReflow detection algorithm already shows the best sensitivity of all methods, we sought to increase its sensitivity to ensure that all SR regions are detected. The bottom row of Table 1 shows the performance that results when it is updated to include a step that dilates the generated segmentation mask with a 5×5 elliptical kernel. Although this step leads to a noticeable decrease in Dice score and IoU, the behavior is expected as the enlarged masks intentionally overstep the bounds of the ground truth SR regions. Importantly, this step leads to a notable sensitivity increase. We believe that mask dilation is warranted for our application because it helps capture SR-adjacent pixel regions that have a manipulated appearance due to their proximity to SR, which can affect the restoration quality due to disruptions in the true color and feature propagation. We explore this topic more in depth in Sec. 4.2. Thus, our final implementation of SpecReFlow detection (SpecReFlow Det.) includes dilation. We thus term the original detection algorithm that does not include dilation as SpecReFlow Det.(-).

**Fig. 6:**
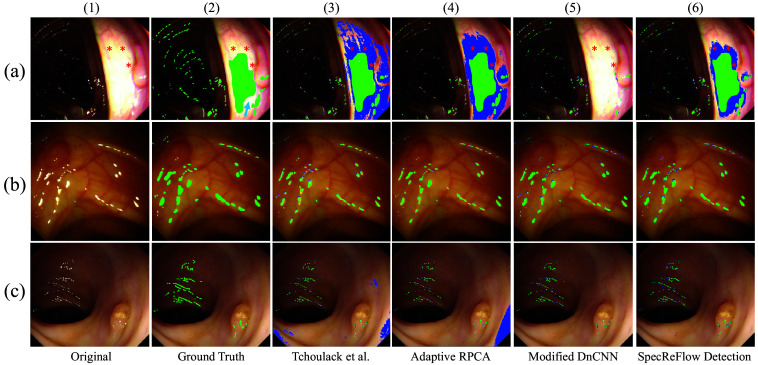
Qualitative comparison of SR detection performance. Performance of algorithms on representative types of SR: (a) a large SR region and SR present in both light and dark regions, (b) medium-sized SR regions scattered across a relatively homogenous region, and (c) small, speckled SR regions scattered in localized regions. The green annotations indicate true positives, and the blue annotations indicate false positives. The red asterisks indicate regions of increased saturation around the central SR region. The blue arrow in the ground truth image (a)(2) indicates a region of high saturation that our detection method picked up but was not indicated in the ground truth.

To assess our results qualitatively, we used a representative subset of the same test dataset from Sec. 3.4.1. The algorithms were evaluated on representative types of SR: (a) a large SR region and SR present in both light and dark regions, (b) medium-sized SR regions scattered across a relatively homogeneous region, and (c) small, speckled SR regions scattered in localized regions. As seen in Fig. 6, the green annotations indicate true positives, and the blue annotations indicate false positives. The red asterisks indicate regions of increased saturation around the central SR region that are not strictly SR but require correction; thus, our algorithm is sensitive to detecting such regions of information loss. SpecReFlow Det. Successfully detects large SR regions [Fig. 6(a)(6)], whereas the modified DnCNN model is unable to [Fig. 6(a)(5)]. In addition, the blue arrow in Fig. 6(a)(2) indicates a location where SR was not labeled in the ground truth image but was still detected with SpecReFlow Det., suggesting that our method is robust to mislabeled annotations.

Considering Figs. 6(c)(3) and 6(c)(4), we see that SpecReFlow Detection [Fig. 6(c)(6)] also led to fewer false positives than the conventional methods (adaptive RPCA and Tchoulack’s algorithm). Furthermore, our method generated a smoother, more continuous mask that captured the entire SR region rather than just the most intense SR regions (as seen in Tchoulack’s algorithm), which can lead to more accurate restorations without random spots of SR. Notably, we observed that the ground truth was manually labeled with a thick marker size, which makes the labeled SR regions appear larger than they are. Thus, some of the false negative regions in Figs. 6(b) and 6(c) (unlabeled) are not actually wrong but appear so due to the overly extensive marker labels in the ground truth. Qualitatively, the “thickness” of the algorithm-marked labels in Fig. 6(c) matches what is expected from the original image.

As mentioned, the final SpecReFlow detection stage includes a marginal dilation step to increase sensitivity and capture adjacent regions that are not strictly SR but that require inpainting. Hence, in some locations, our detection mask covers a larger area than the “ground truth” SR. However, some of these additional regions are warranted: SpecReFlow Det. can accurately identify highly saturated regions (red asterisks) that are adjacent to the main SR region and contribute to information loss, as demonstrated by their coverage indicated with blue regions in Fig. 6(a)(6). Furthermore, our method does not contribute false positives that still contain relevant information, unlike the blue areas in Figs. 6(a)(3) and 6(a)(4) that mask regions with useful spatial information. This exemplifies the effectiveness of our method in identifying only highly saturated regions of interest with information loss.

We also performed an ablation study to assess the effectiveness of each of the SR detection pipeline components: preprocessing, deep learning (Light U-net), and thresholding. The input to each run comprised a raw or preprocessed RGB image, and the output was an SR mask. We analyzed the time and improvement in quantitative detection metrics as each component was systematically added or removed from the pipeline. The results can be observed in Table 2. Notably, the impact of preprocessing on quality performance metrics (Dice and IoU) could not be assessed as preprocessing alone is not a form of detection and yields no detected mask output.

**Table 2:** Quantitative ablation study of SR detection performance.

Detection component(s)	Dice ↑	IoU ↑	Sensitivity ↑	Time (ms) ↓
Raw image				
Light U-net	0.6892	0.5560	0.7614	9.39
Thresholding	0.2945	0.2087	0.8367	**0.51**
Light U-net + thresholding	0.3020	0.2117	**0.9462**	9.83
Preprocessed image				
Light U-net	0.7881	0.6837	0.7636	10.76
Thresholding	0.5540	0.4047	0.4247	1.64
Light U-net + thresholding	**0.8285**	**0.7158**	0.8158	10.92

Note: bold values highlight the best performing algorithm for each metric.

Using only raw images as input, simple thresholding takes less than a millisecond (0.51 ms) of runtime on average. We found that using the Light U-net model alone takes significantly longer—9.39 ms of runtime on average—but improves the Dice score and IoU metrics by nearly 2.03× compared with only using thresholding. Thresholding, however, yields better sensitivity than only using Light U-net (0.8367 versus 0.7614). Furthermore, when comparing the results of using Light U-net + thresholding versus using only thresholding, we surprisingly find the Dice and IoU metrics to be similarly low, which can be explained by the high amount of detection error contributed by the simple thresholding component.

Image preprocessing adds 1.20 ms of runtime on average but makes significant improvements across all detection metrics: Light U-net metrics improved by 1.13× on average, thresholding metrics improved by 1.44× on average, and Light U-net + thresholding metrics improved by 2.37× on average. When comparing the results of using preprocessed images with the Light U-net + thresholding method versus using only thresholding, we find a significant 1.63× improvement in Dice score and IoU metrics—a major difference from when only raw images were used as input.

Thresholding on preprocessed images is significantly worse than Light U-net alone in terms of sensitivity (0.4247 versus 0.7636). Although thresholding does seem to have superior sensitivity to Light U-net when using raw images, it is important to note the significantly lower Dice and IoU metrics produced by thresholding for both raw and preprocessed images. This can be explained by the fact that, although thresholding detects more of the “true” SR region, it does so by also detecting many more non-SR regions (low specificity), which will cause the restoration to be more difficult.

Although mask dilation is the last step of the SpecReFlow detection stage, we chose not to include it in our ablation study for the following reasons. (1) We wanted to measure detection performance using Dice and IoU metrics before optimizing for sensitivity, so the predicted SR mask is as close to the ground truth as possible. (2) We wanted the restored SR region to seamlessly integrate with the context of its surroundings, which makes the mask dilation step more relevant for the SpecReFlow restoration stage rather than for the detection stage on which the ablation study focuses.

Overall, the results of the ablation study of our detection pipeline components show that each component adds minimal runtime to SR detection while contributing significant improvements in all detection metrics.

**Fig. 7:**
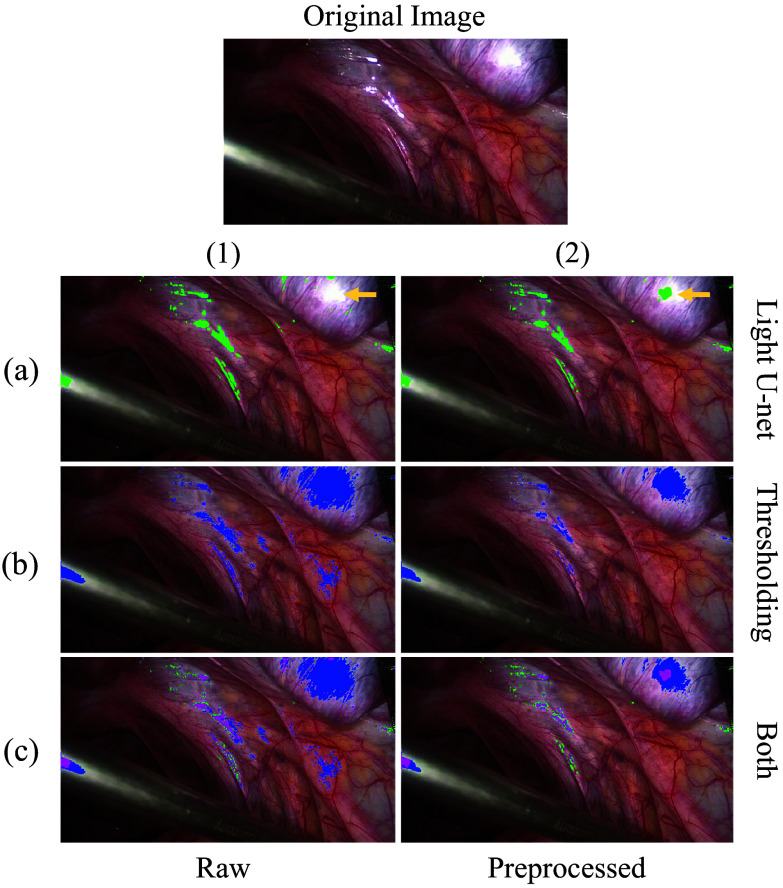
Qualitative ablation study of our detection pipeline components. To visually explain the contributions of each component of our detection pipeline, masks generated from combinations of components are shown. The green mask indicates predictions from only using Light U-net, the blue mask indicates predictions from only using thresholding, and the magenta mask indicates intersections between the green and blue masks. As expected, thresholding detects large SR regions well, and the Light U-net detects smaller regions well.

Figure 7 corroborates the results of our ablation study. Figure 7(a)(1) shows that the deep learning model works poorly in detecting larger SR regions (indicated by the gold arrow), but it performs well in covering all of the relatively smaller SR regions. Switching to using preprocessed images, Fig. 7(a)(2) shows how the model can recognize a hotspot in the large SR region, indicating an improvement in detection.

In Fig. 7(b)(1), we see that thresholding alone of raw images includes significant amounts of unsaturated pixels, which makes the restoration task more difficult. By applying preprocessing [Fig. 7(b)(2)], thresholding works much better to only detect saturated regions with information loss; however, the smaller SR regions are weakly detected. By combining preprocessing, Light U-net, and thresholding [Fig. 7(c)(2)], it can be seen that the Light U-net detects the smaller SR regions where thresholding fails (seen by the green mask enveloping the magenta mask), and thresholding detects the larger SR regions where the Light U-net fails (seen by the blue mask enveloping the magenta mask), as the magenta mask represents the intersection between the blue and green mask. Thus, both small and large SR regions are fully detected, which is reflected well in the quantitative detection metrics.

### 4.2 SR Restoration

**Fig. 8:**
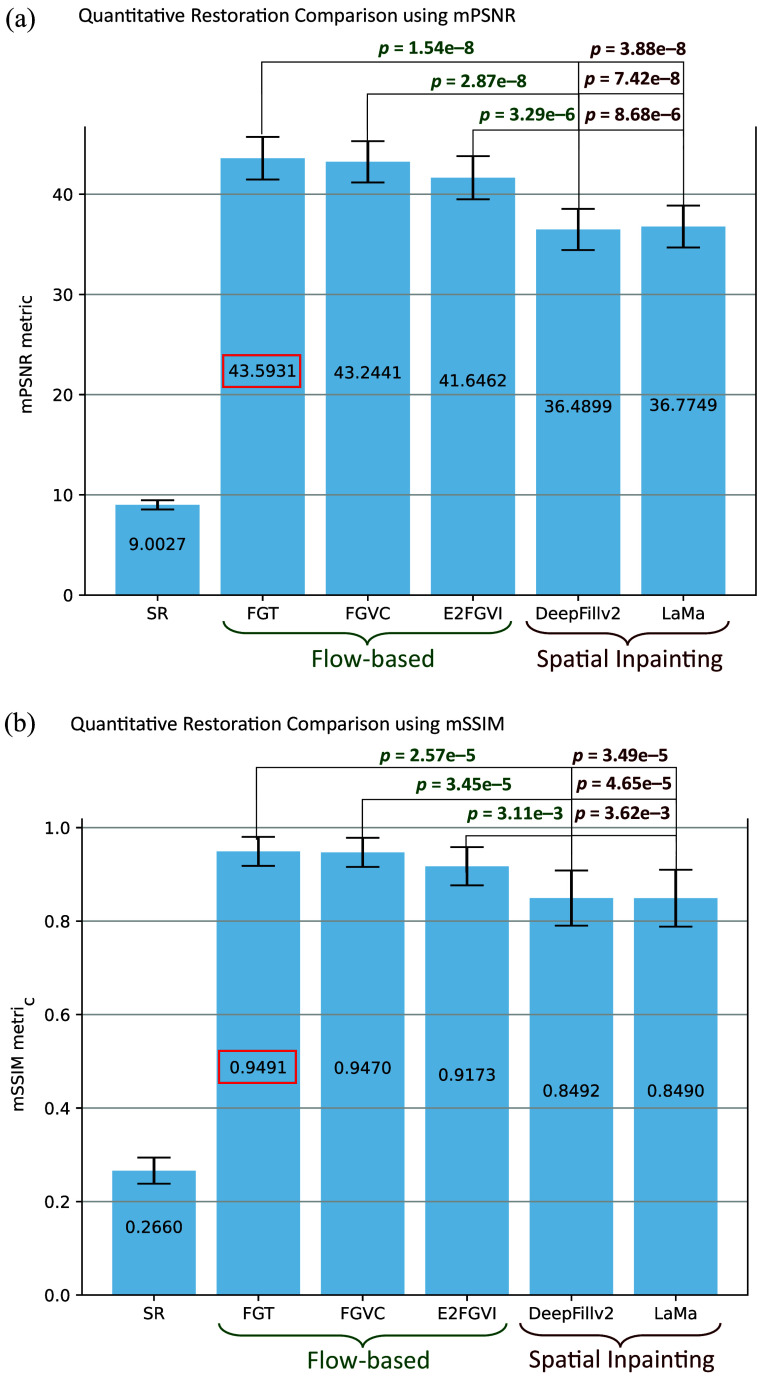
Visual quantitative comparison of SR restoration methods. Restoration (a) mPSNR and (b) mSSIM are measured for 13 independent SR restoration trials. The following restoration algorithms and baselines are compared: an SR unrestored image as the baseline, optical flow-based methods such as FGT, FGVC, and E2FGVI, and single-frame inpainting methods such as DeepFillv2 and LaMa. The p-values associated with two-tailed, two-sample T-tests of means comparing each flow-based algorithm with the inpainting options are displayed above the bars.

Figure 8 compares the mPSNR and mSSIM performance of several restoration algorithms. In general, the flow-based algorithms (FGT, FGVC, and E2FGVI) performed better than single-frame inpainting methods (DeepFillv2 and LaMa) across both metrics. As expected, FGT outperforms FGVC because it incorporates optimizations by design. All algorithms outperform the baseline of not restoring the region at all. Importantly, the differences are statistically significant.

From visual observation, FGT and FGVC did not have overlapping error bars with those of the single-frame inpainting methods, suggesting that those two algorithms performed the restoration task significantly better than that of the single-frame inpainting methods. The end-to-end method (i.e., E2FGVI) had overlapping bars with all other methods, showing weak evidence to support a significant difference.

In both bar charts, comparing the restoration methods within their classes results in overlapped bars, showing strong evidence that there was not a significant difference. To analyze the relationship quantitatively, for each restoration metric (i.e., mPSNR and mSSIM), we conducted six combinations of two-tailed two-sample t-tests for means with the baseline being a spatial inpainting algorithm and the “after” being a flow-based algorithm. The resulting p-values are shown in Fig. 8. Because the null hypothesis is that there is no significant difference between a selected spatial inpainting algorithm and a flow-based algorithm, with all resulting p-values much less than 0.01, we find strong evidence that there is a significant difference and visually observe that flow-based algorithms have significantly higher restoration metrics than do spatial inpainting algorithms. Indeed, FGT has the best performance of all (highest mPSR, mSSIM, and most statistically significant difference from conventional methods) and was therefore selected as the restoration algorithm of choice for SpecReFlow.

**Fig. 9:**
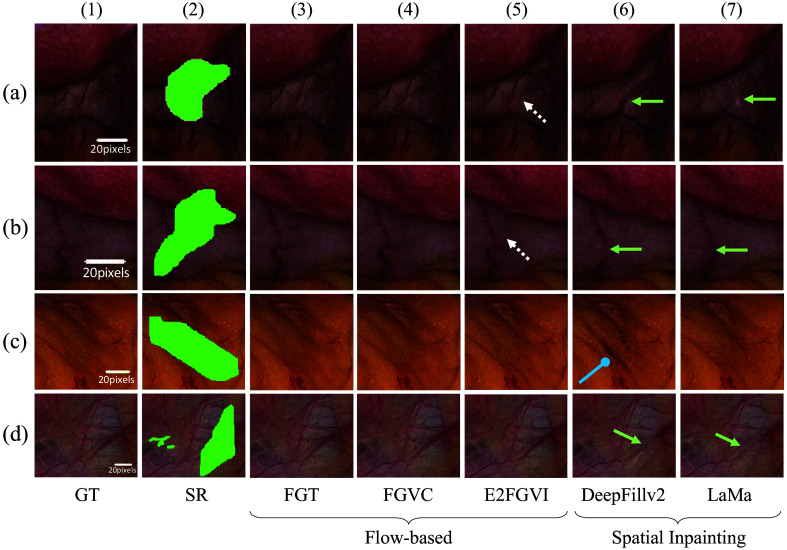
Qualitative comparison of SR restoration performance. Qualitative comparison of flow-based and single-frame restoration algorithms. Four representative SR masks (green) are applied to qualitatively test restoration in (a)–(d). DeepFillv2 and LaMa fail to restore key structural features such as blood vessels (green arrows), with distortions or omissions evident. DeepFillv2 also struggles with larger areas, creating darker pixels and artifacts (blue rounded arrow). In contrast, optical flow-based algorithms like FGT, FGVC, and E2FGVI preserve structural integrity and color accuracy. However, E2FGVI shows undesirable smoothing (white dashed arrows), affecting its quantitative scores. Pixel-wise scale bars illustrate relative resolution between samples.

**Fig. 10:**
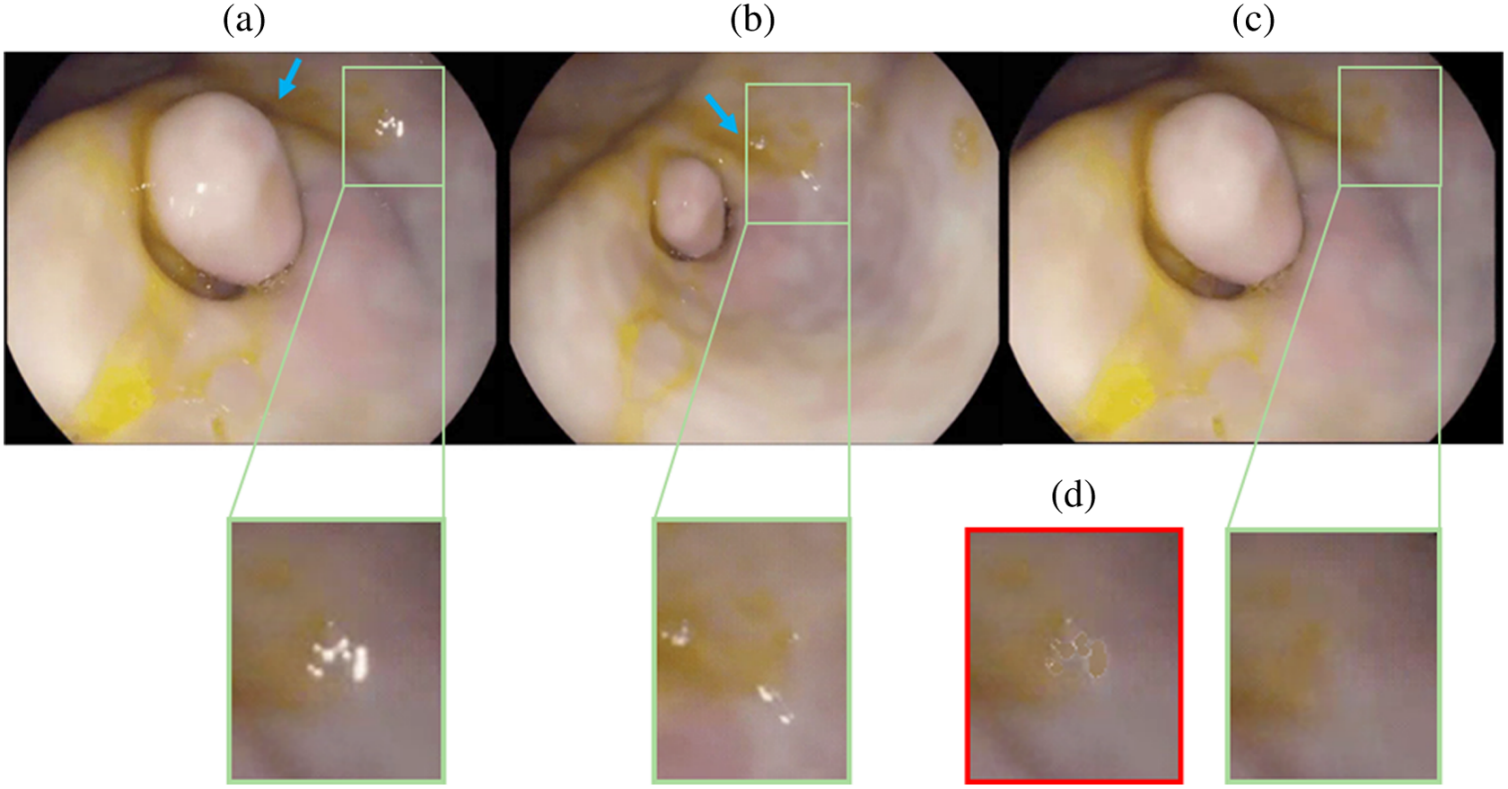
Restoration under different lighting conditions. Green insets show enlarged versions of each picture covering the region of intended restoration. The blue arrows emphasize regions that have significantly different lighting across frames. Panel (a) shows a frame with an SR region. Panel (b) is a later frame with target pixels that are not obscured by SR; however, it has different lighting so direct replacement is not reasonable. Panel (c) shows the restored version of panel (a) that is free from SR artifacts with appropriate consideration of lighting changes. Panel (d) represents the baseline algorithm for restoration using homography transform for direct pixel replacement, which leads to color and lighting inconsistencies (Video 1, MP4, 1.02 MB [URL: https://doi.org/10.1117/1.JMI.11.2.024012.s1]).

Our qualitative analysis also supports the superior performance of flow-based restoration algorithms compared with single-frame counterparts. For example, in Fig. 9, both DeepFillv2 and LaMa fail to accurately restore structurally important features such as blood vessels (indicated by the green arrows), which appear distorted in some cases and not at all in others. Furthermore, we observed that DeepFillv2 struggled to correctly inpaint larger areas such as in Fig. 9(c)(6), which resulted in darker pixels and more artifacts (indicated by the rounded blue arrow). Compared with the single-frame inpainting restoration algorithms, optical flow-based algorithms (FGT, FGVC, and E2FGVI) performed favorably: not only do structural features appear in their correct likeness, but the colors are highly matched as well. E2FGVI, however, demonstrated undesirable smoothing (indicated by dashed white arrows), which may be responsible for its lower performance on the quantitative metrics. Overall, the qualitative results reflect the conclusions drawn from the quantitative analysis. Note that Fig. 9 contains pixel-wise scale bars that compare the relative resolution among samples, as physical measurements were not available. In Fig. 10, we demonstrate that our algorithm works in different lighting conditions, in which direct replacement is not as accurate as a restoration option. A demonstration video of the full SpecReFlow algorithm in action is provided as Video 1.

## 5 Conclusion

The need to improve the visualization of internal structures using endoscopy video by eliminating SR image artifacts motivates a complete and systematic approach to SR detection and restoration. To this end, we have created a complete solution for removing SR artifacts from endoscopy videos, called SpecReFlow. Our approach combines image preprocessing for contrast enhancement, deep learning, and conventional methods for complete SR detection, and it uses transformer and optical flow-based methods to leverage both temporal and spatial data for SR restoration.

SpecReFlow addresses the limitations of conventional detection algorithms by being able to detect all types of SR regions while doing so without the time-consuming, inefficient, and ungeneralizable empirical parameter setting needed in conventional methods. Using flow-based restoration algorithms, our approach not only removes the need to predict features and colors directly, as done in other spatial inpainting methods, but also fixes the issue of lighting inconsistencies from direct pixel replacement in multiview restoration methods by operating in the color gradient domain. Ultimately, we showed how incorporating temporal information from adjacent slides leads to superior restorations compared with directly inpainting the SR region. In addition, we proposed two new metrics, mPSNR and mSSIM, to evaluate the quality of SR restoration more accurately than existing metrics.

Although the algorithm itself works well, a particularly challenging aspect of this work is the memory requirement. Although the vision transformer in the restoration algorithm can process relatively many frames at once, the virtual memory overhead is cost inefficient and not scalable to longer-duration endoscopy videos. Future investigation of memory-efficient transformer architectures, efficient loading of data from CPU to GPU, or a chunking method to encode new temporal information can drastically increase the restoration performance on longer duration frames. Another edge case is that, when both the camera/lighting and tissue are truly stationary across many frames, the calculated optical flow (which would be minimal because there is no movement) could be exploited to remove redundant frames and allow for more efficient processing. Once the set of frames is de-duplicated and the remaining frame is restored using our proposed algorithm, the result can be broadcasted to the initial set of duplicate frames. We leave the implementation of this memory-saving method to future work.

By providing a reliable and efficient solution for SR artifact removal in endoscopy videos, SpecReFlow can greatly improve diagnostic accuracy and efficiency, facilitating clearer visualization for early detection and management of diseases. Furthermore, we hope that our approach can be extended to other imaging modalities and environments to remove image artifacts in an efficient and reliable manner.

One promising future use case for SpecReFlow Det. is to generate accurate labels for other endoscopy datasets and build a database of annotated SRs that can be used to train a better detection network. Future work can be done with this new dataset to encode the predictions of a multicomponent detection algorithm into a singular more efficient and well-trained model architecture that performs just as well as, if not better than, our current detection algorithm.

## Supplementary Material



## Data Availability

The code, supporting documentation, and publicly available datasets for this paper can be found at https://osf.io/brmg9/
